# Tuina for peripherally-induced neuropathic pain: A review of analgesic mechanism

**DOI:** 10.3389/fnins.2022.1096734

**Published:** 2022-12-22

**Authors:** Zhi-Feng Liu, Hou-Rong Wang, Tian-Yuan Yu, Ying-Qi Zhang, Yi Jiao, Xi-You Wang

**Affiliations:** ^1^Department of Tuina and Pain Management, Dongzhimen Hospital, Beijing University of Chinese Medicine, Beijing, China; ^2^School of Acupuncture-Moxibustion and Tuina, Beijing University of Chinese Medicine, Beijing, China; ^3^Clinical Medical College, Beijing University of Chinese Medicine, Beijing, China

**Keywords:** neuropathic pain, tuina, peripheral nerve injury, inflammation, glial cells, brain function

## Abstract

Peripherally-induced neuropathic pain (pNP) is a kind of NP that is common, frequent, and difficult to treat. Tuina, also known as massage and manual therapy, has been used to treat pain in China for thousands of years. It has been clinically proven to be effective in the treatment of pNP caused by cervical spondylosis, lumbar disc herniation, etc. However, its analgesic mechanism is still not clear and has been the focus of research. In this review, we summarize the existing research progress, so as to provide guidance for clinical and basic studies. The analgesic mechanism of tuina is mainly manifested in suppressing peripheral inflammation by regulating the TLR4 pathway and miRNA, modulating ion channels (such as P2X3 and piezo), inhibiting the activation of glial cells, and adjusting the brain functional alterations. Overall, tuina has an analgesic effect by acting on different levels of targets, and it is an effective therapy for the treatment of pNP. It is necessary to continue to study the mechanism of tuina analgesia.

## 1. Introduction

Neuropathic pain (NP) is a pain directly caused by injury or disease of the somatosensory nervous system (Jensen et al., 2011; Colloca et al., 2017), which can be caused by injury of the nerve, spinal cord, or brain, as well as diabetes, herpes zoster, etc. (Alles and Smith, 2018). According to the injury or anatomical location, NP can be divided into peripherally-induced neuropathic pain (pNP) and centrally-induced neuropathic pain. pNP is the most common with a prevalence rate of 6.9∼10% (Finnerup et al., 2015). A recent survey shows that the prevalence rate in China is 29.53∼31.54% (Yongjun et al., 2020). pNP is a risk factor leading to sleep disorders, anxiety, depression, and suicide, and seriously affects the quality of human life (Petrosky et al., 2018). The treatment of pNP depends largely on pharmacology, but some studies have shown that medications are insufficiently effective and not innocuous (Finnerup et al., 2015, 2018; Meacham et al., 2017), while traditional Chinese medicine (TCM) therapies are effective in relieving pain with little side effects.

As one of the characteristic therapies of TCM, tuina has been used in China for thousands of years to treat pain. Under the guidance of TCM and western medicine anatomy and pathology, tuina acts on the body surface by various manipulations, such as rubbing, kneading, and pressing, to regulate the physiological and pathological state, so as to treat diseases (Wang et al., 2008). A survey in China showed that 66.8% of patients with chronic pain chose tuina or cupping (Yongjun et al., 2020). Pain is an advantageous symptom for tuina treatment, especially pNP caused by cervical spondylosis radiculopathy (CSR) (Cao et al., 2021; Aboagye et al., 2022), lumbar disc herniation (LDH) (Mo et al., 2019; Zhou et al., 2022), etc. A meta analysis showed that tuina and acupuncture were more effective than traction and TCM in the treatment of LDH (Mo et al., 2019). Through a clinical trial, Cao et al. (2021) showed that both three-dimensional balanced tuina therapy and traditional tuina can effectively relieve pain in patients with CSR. Based on the clinical advantages of tuina analgesia, its mechanism is always the focus of research, and some progress has been made in recent years. Hence, this review aims to summarize the existing research progress on the analgesic mechanism of tuina.

## 2. Tuina exerts analgesic effects by suppressing peripheral inflammation

Inflammation is the key to peripheral and central sensitization and plays an important role in the initiation and maintenance of pNP (Ellis and Bennett, 2013). Peripheral nerve injury (PNI) leads to a local inflammatory response, activates related inflammatory pathways in mast cells and macrophages to release inflammatory mediators, which enhance the sensitivity of nociceptive receptors. Some studies have shown that tuina can reduce the levels of inflammatory factors in blood and dorsal root ganglion (DRG), such as tumor necrosis factor-α (TNF-α) and interleukins (IL-6 and IL-1β) (Tao et al., 2017; Yang et al., 2020; Yao et al., 2022a), an effect that is mainly achieved by regulating related pathways.

Toll-like receptor 4 (TLR4) pathway, one of the key inflammatory signal transduction pathways, exerts an important role in mediating pNP (Liu et al., 2012; Li et al., 2019). After nerve injury, TLR4 pathway is activated in sensory neurons, which induces the expression of proinflammatory cytokines through two pathways, one of which is the MyD88 pathway (Liu et al., 2012). TLR4 binds to MyD88 and activates interleukin 1 receptor kinase (IRAK1), which binds to TRAF6 to form a receptor complex, and then activates mitogen-activated protein kinase (MAPK) and NF-κB signaling pathways. Rodent studies have shown that activation of TLR4 pathway in DRG or spinal dorsal horn leads to hyperalgesia in NP models (Agalave et al., 2014; Lin et al., 2015). Spinal nerve ligation (SNL) is a model for simulating spinal nerve injury, which shows typical pNP manifestations, such as spontaneous pain, thermal hyperalgesia, mechanical hyperalgesia, etc. (Seto et al., 2021). A study showed that 14 days after modeling, the mRNA expression of TLR4, IRAK1, and TRAF6 were significantly increased in SNL rats, and the expression levels of TNF-α and IL-6 also increased. After 14 days of tuina intervention, including pointing, stroking, and kneading methods, the levels of TLR4, IRAK1, TRAF6, TNF-α, and IL-6 decreased (Wang et al., 2020), which suggests that tuina may reduce the expression of inflammatory factors by inhibiting TLR4 pathways. He et al. (2021) also found the increased levels of TLR4, MyD88, NF-κB p65, IL-6, IL-1β, and TNF-α in LDH rats. And after giving the intervention of pressing, kneading, and pushing manipulations, these levels were decreased. Furthermore, the levels decreased after injection of TLR4 inhibitors in the model group and increased after administration of TLR4 activator in the tuina group. Wang et al. (2022), using RNA-Seq, found that one-time tuina intervention could regulate TLR and NF-κB signaling pathways in minor chronic constriction injury (CCI) rats. It is suggested that tuina can reduce the expression of inflammatory factors by inhibiting the activation of TLR4/NF-κB signaling pathways, thereby achieving anti-inflammatory and analgesic effects. However, the specific mechanism of how tuina regulates these pathways remains unclear.

MicroRNAs (miRNAs) are the master switch linking nerve injury, pain, and inflammation, which play an important regulatory role in pain signal transduction (Sakai and Suzuki, 2014; Sommer et al., 2018; Gada et al., 2021). In recent years, an increasing number of studies found that miRNAs are involved in the occurrence and development of NP after nerve injury. Peng et al. (2017) found that miR-183 can control more than 80% of NP regulatory genes and regulate mechanical sensitivity and mechanical pain abnormalities, proving that miRNA can inhibit NP transduction. Sakai et al. (2017) found that the expression of all cluster members of miR-17-92 in DRG was upregulated after nerve injury. In a bilateral CCI rat model, the expression of miR-341 in DRG was upregulated, while miR-203, miR-181a-1*, and miR-541* were downregulated (Li et al., 2013). MiRNAs mediate their role in NP through signal transduction pathways, such as TLR4, NF-κB, NLRP3, etc. (Gada et al., 2021). Jing et al. (2022) found that upregulating the expression of miR-146a in rats with LDH could reduce the activity of the TLR4 signaling pathway, thus relieving pain. Through high-throughput sequencing technology, Yao et al. (2022b) found that there were 19 expressed miRNAs that were related to inflammation in chronic compression of dorsal root ganglia (CCD) rats treated with pressing and kneading “Weizhong” (BL40) compared to CCD model rats. Furthermore, miR-547-3p may be a key target of tuina analgesia, since its overexpression can reduce the expression of Map4k4, and then inhibit the expression of IκBα, p-IκBα, p65, and p-p65 in the NF-κB signaling pathway, suggesting that tuina can exert an analgesic effect by targeting Map4k4 *via* miR-547-3p.

## 3. Tuina exerts analgesic effects by modulating the peripheral ion channels

The transmission and processing of pain signals heavily depend on the activity of ion channels. In nerve injury, dysregulation of ion channel expression leads to increased neuronal excitability, which is the basis of pNP (Waxman and Zamponi, 2014). Ion channels include voltage-gated, ligand-gated, and mechanosensitive ion channels (MSC). P2 purinergic receptors, divided into P2X ion ligand-gated receptor and P2Y metabolic G protein-coupled receptor, are a class of ligand-gated receptors activated by extracellular ATP and its metabolites. P2X3 receptor is selectively expressed in primary sensory neurons and is involved in pain signal transduction. It was found that P2X3 receptor expression was upregulated in DRG neurons after nerve injury (Krajewski, 2020). It was found that tuina can inhibit the expression of P2X3 receptor in DRG of CCI model rats (Chen et al., 2022) and LDH model rats (Lin et al., 2017), and it can also reduce the amplitude of inward current in P2X3 channel (Chen et al., 2022), which proved that tuina can downregulate the expression of P2X3 as well as the degree of channal opening in DRG. MSC are ion channels that convert the mechanical stimulation sensed by the cell membrane into bioelectric or biochemical signals. Piezo, including Piezo1 and Piezo2, plays an important role in the sensation and transmission of mechanical stimuli such as pain and touch (Kim et al., 2012). Tuina that included clockwise pressing and rubbing can increase the expression of Piezo2 and decrease the expression of Piezo1 in CCD rats (Song et al., 2018). As a mechanical stimulation, tuina acts on the skin through manipulation, so tuina may play a role by activating MSC.

## 4. Tuina exerts analgesic effects by inhibiting the activation of glial cells in spinal cord

Glial cells, including microglia, astrocytes, and oligodendrocytes, play a critical role in the production and maintenance of pNP (Ji et al., 2014; Inoue and Tsuda, 2018). After nerve injury, glial cells are activated and the activated state is mainly manifested by glial reaction, upregulation of glial receptor, proliferation, etc. (Ji et al., 2013). Glial reaction refers to the upregulation of markers and morphological changes of glial cells. Wu et al. (2022) found the expression of microglia and astrocyte gene in the spinal dorsal horn of the CCI model rats was significantly higher than that of control rats, and the expression of astrocyte marker GFAP, microglia marker Iba-1, and M1 receptor CD68 increased significantly. In contrast, the expression of activated glial cell genes as well as the glial markers and receptors decreased significantly after tuina intervention. Morphological changes are the most direct manifestation of microglial activation (Nair et al., 2019). Partially activated or inactivated microglia are branched. After activation, the cytosol becomes larger, processes are shortened, and become round or rod-shaped. Mo et al. (2020) observed that the microglia in rats with sciatic nerve injury had short processes and enlarged cytoplasm, whereas the microglia after tuina treatment *via* three-method (pressing, plucking, and kneading manipulations) and three-acupoint (BL37, GB34, and BL57) treatment had longer protrusions and smaller cytoplasm, demonstrating that tuina could inhibit the activation of microglia.

In addition, activation of intracellular signaling pathways, such as MAPK, are also a manifestation of glial cell activation (Ji et al., 2013). MAPK, including extracellular signal-regulated kinase (ERK), p38, and c-Jun N-terminal kinase (JNK), can transduce extracellular stimulation into intracellular transcriptional and post-translational effects. p38 plays a key role in microglia signal transduction of pNP and is a valuable target for the treatment of pNP (Ji and Suter, 2007). Activation of p38 was found in spinal cord microglia of SNL (Ji and Suter, 2007), spinal cord injury (Hains and Waxman, 2006), and ventral root lesion (Xu et al., 2007). In a CCI model, Wei et al. (2018) found that the expression of phosphorylated p38 and IL-1β in the spinal cord increased, whereas these same factors were decreased after tuina intervention, suggesting that tuina may inhibit the activation of microglia by regulating the MAPK pathway.

## 5. Tuina exerts analgesic effects by adjusting the functional alterations in brain

The brain, the main site of pain production, is critical in the development and relief of pNP. After the nociceptive stimulation passes through the periphery and spinal cord, it will be further transmitted to the brainstem network structure, thalamic and hypothalamic nucleus, and then projected to the cerebral cortex and limbic system to produce pain sensation and pain response (Meacham et al., 2017). Pain is a complex multi-channel subjective experience, including sensory and emotion. After the nociceptive information is uploaded to the brain, it will be divided into several parallel pathways, in which the somatosensory cortex is involved in the sensory aspect of pain processing, and anterior cingulate cortex (ACC) is involved in the emotional aspect of pain processing (Weizman et al., 2018). It was found that after tuina intervention, the amplitude of low-frequency fluctuation (ALFF) value of the somatosensory cortex increased, indicating that tuina can participate in pain management by activating the somatosensory cortex (Xing et al., 2021).

When the body receives a painful stimulus, a number of cortical and subcortical brain areas are activated together, and these brain areas are linked together to form a brain network (Legrain et al., 2011). It has been shown that some brain regions of the pain-related network are involved in the generation and transmission of pain (Giesecke et al., 2004; Shi et al., 2021). For example, the marginal and striatal structures, including anterior cingulate cortex (ACC), superior frontal gyrus, medial thalamus, and anterior insular cortex, show enhanced responses in patients with pNP caused by diabetic peripheral nerve deformation (Tseng et al., 2013). The primary sensory cortex, cingulate gyrus, amygdala, thalamus, and insula are activated in patients with chronic low back pain (cLBP) (Sharma et al., 2011). Yuan et al. (2015) found that spinal manipulation can effectively reduce the VAS score of patients with LDH. Furthermore, brain functional magnetic resonance imaging showed that the brain functional activity was mainly inhibited, and the inhibitory area was mainly located in the right prefrontal lobe and cerebellum, indicating that tuina may relieve pain by inhibiting the functional activities of the frontal lobe and cerebellum. Tan et al. (2020) found that the brain activity in the right parahippocampal gyrus, the right dorsolateral prefrontal cortex, and the left precuneus was significantly increased after one time of spinal manipulative treatment, and the brain activity in the posterior cingulate cortex (PCC) and right inferior frontal gyrus was significantly increased after six times of treatment.

Cerebral functional alterations depend on changes in neurotransmitter levels (Tabassum et al., 2020). Huo et al., 2022a,b found that after one time of tuina treatment in patients with cLBP, the level of *N*-acetylaspartic acid (NAA) in the PCC increased and Glutamate complex (Glx) decreased, and the level of Glx3 in the PCC decreased after six times of tuina treatment. Existing studies have shown also decreased NAA levels in multiple brain regions in patients with pNP. For example, NAA levels were reduced in the ACC of cLBP patients (Zhao et al., 2017), and also decreased in the PCC of patients with trigeminal neuralgia (Fayed et al., 2014). NAA is the main marker of neuronal integrity, the decrease of NAA levels suggests neuronal dysfunction or injury (Moffett et al., 2013; Zhang et al., 2013). Tuina can increase the level of NAA, indicating that tuina participates in analgesia by enhancing neuronal repair. Glx, one of the main excitatory neurotransmitters in mammalian nervous system, plays an important role in neuronal regulation. High concentrations of Glx can induce neuronal hyperexcitability and sustained activation, and it can also increase oxidative stress, causing neuronal injury and death (Zhang et al., 2013). Tuina can reduce the level of Glx, indicating that it can exert analgesic effects by reducing neuronal hyperexcitability and reduce oxidative stress. Glx is also involved in mood regulation. A study has shown that tuina can relieve the negative emotions in pain patients (Xu et al., 2022), suggesting that tuina may reduce pain sensitivity by improving negative emotions. Zhang et al. (2014) found that the levels of gama amino-butyrlc-acid (GABA) and GABAAR in the periaqueductal gray and rostral ventromedial medulla of CCI rats increased after pressing and kneading the “Huantiao” (GB30). GABA can inhibit the transmission of nociceptive information. After nerve injury, the activity of GABA is decreased, therefore resulting in an inability to exert GABA’s effect of central inhibition, thus inducing abnormal pain and hyperalgesia (Gwak and Hulsebosch, 2011). So tuina can upregulate the expression of GABA and GABAAR, and then reduce the transmission of harmful information.

## 6. Conclusion

**FIGURE 1:**
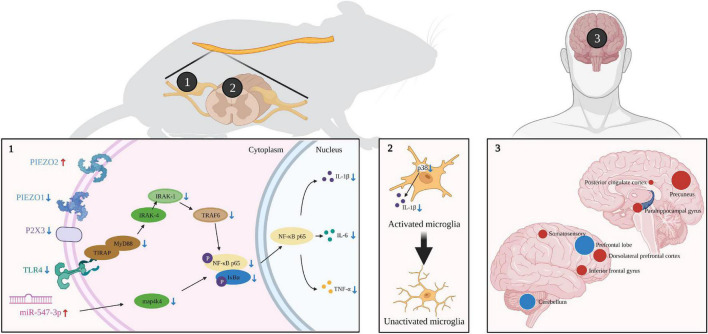
The analgesic mechanism of tuina [images with permission created with BioRender. https://biorender.com (2022)].

Tuina treats pNP by acting on different levels of targets (Figure 1). At the peripheral level, tuina can inhibit the expression of inflammatory factors, such as IL-1β, IL-6, and TNF-α by inhibiting the TLR4/NF-κB pathways in DRG, which may be achieved by upregulating miR-547-3p. Tuina can also modulate the ligand-gated and mechanosensitive ion channels, as evidenced by the fact that it can downregulate the expression of the P2X3 receptor and Piezo1, and upregulate the expression of Piezo2. At the central level, tuina can inhibit the activation of microglia and astrocytes by changing the level of markers and cell morphology, as well as the activation of MAPK pathway in microglia. In addition, tuina can adjust the functional alterations in brain, mainly by inhibiting the activity of the prefrontal lobe and cerebellum, enhancing the activity of the parahippocampal gyrus, dorsolateral prefrontal cortex, precuneus, PCC, etc., and regulating the expression of neurotransmitters, such as NAA, Glx3, and GABA.

Although some progress has been made on elucidating the mechanism of tuina analgesia, there are still some limitations. First, lack of in-depth research on each part. For example, it has been showed that tuina can inhibit the activation of glial cells and change the cerebral function, but the specific pathway or target is not clear enough. Next, lack of connection among the parts. Pain is a complex and multi-channel subjective experience, which requires a series of reactions from the afferent of nociceptive stimulation to the generation of pain. Furthermore, a series of reactions is also required from the action of tuina on the skin to the relief of pain, so the analgesic mechanism should be a dynamic process. Most of the existing studies are independent of each part, and there is a lack of connection between each part. What’s more, there is a lack of comprehensive research, such as an immediate analgesic mechanism. Clinical experience shows that the pain can be obviously be relieved and last for a period of time after one tuina treatment, but the mechanism is not clear.

In conclusion, tuina, as a characteristic TCM therapy, can effectively treat pNP, and its analgesic mechanism includes many parts. Elucidating the analgesic mechanism of tuina is not only conductive to the treatment of pain, but may also lay a foundation for the development of tuina as well as TCM. Therefore, it is extremely important to continue studying the analgesic mechanism of tuina.

## Author contributions

Z-FL drafted the manuscript. H-RW drew the picture. Y-QZ and YJ collected the literature. Z-FL, T-YY, and X-YW put forward the idea. All authors contributed to the manuscript and approved the submitted version.
